# Case Report: Dupilumab-associated psoriasiform eruption followed by depigmentation in a patient with prurigo nodularis and Vogt-Koyanagi-Harada disease

**DOI:** 10.3389/fimmu.2026.1991039

**Published:** 2026-10-21

**Authors:** Miku Otsuka, Emi Sato, Shiori Katayama, Shinichi Imafuku

**Affiliations:** Department of Dermatology, Faculty of Medicine, Fukuoka University, Fukuoka, Fukuoka, Japan

**Keywords:** atopic dermatitis, depigmentation, dupilumab, immune shift, prurigo nodularis, psoriasiform eruption, vitiligo, Vogt-Koyanagi-Harada disease

## Abstract

Dupilumab blocks interleukin (IL)-4 and IL-13 signaling and is effective for type 2 inflammatory diseases, including atopic dermatitis and prurigo nodularis. Paradoxical psoriasiform eruptions and, less commonly, new-onset or worsening vitiligo have been reported during treatment, suggesting that selective type 2 blockade can alter the cutaneous immune balance in susceptible individuals. We describe a 70-year-old Japanese man with long-standing atopic dermatitis that had evolved into prurigo nodularis and a medical history of Vogt-Koyanagi-Harada (VKH) disease, a melanocyte-directed autoimmune disorder. At follow-up approximately two weeks after a single 600-mg loading dose of dupilumab, the patient presented with a rapidly generalized psoriasiform eruption in a striking distribution that largely spared pre-existing pruriginous nodules. Dupilumab was discontinued, and a biopsy of a new plaque supported a treated or drug-associated psoriasiform inflammatory eruption. Oral cyclosporine was initiated, with improvement of the eruption. Approximately six months after dupilumab administration, marked depigmentation was documented at sites corresponding to the preceding psoriasiform eruption. The pigmentary change was clinically considered severe post-inflammatory hypopigmentation; however, vitiligo remained an important alternative diagnosis because of the patient’s VKH background. The temporal sequence raises the hypothesis that selective type 2 blockade may alter cutaneous immune balance in patients with pre-existing melanocyte-directed autoimmunity, but it does not establish dupilumab-induced vitiligo or systemic VKH reactivation. Broader pathway-directed approaches, including modulation of the IFN-γ–JAK–STAT axis, warrant further investigation in such patients.

## 1 Introduction

Dupilumab is a monoclonal antibody against the interleukin (IL)-4 receptor alpha subunit that inhibits IL-4 and IL-13 signaling and is effective for atopic dermatitis and prurigo nodularis. Psoriasis and psoriasiform eruptions are uncommon paradoxical reactions during dupilumab therapy; a proposed mechanism is release of the IL-23/IL-17 axis from type 2-mediated restraint (1).

Vitiligo is characterized by melanocyte-directed cytotoxic T cells and IFN-γ-dependent CXCL9/CXCL10-CXCR3 signaling, with tissue-resident memory programs contributing to persistence (2, 3). New-onset and worsening vitiligo have been reported during dupilumab treatment, although the available evidence does not establish a causal drug effect (4, 5).

Vogt-Koyanagi-Harada (VKH) disease is a systemic autoimmune disorder targeting melanocyte-associated antigens and can cause vitiligo and poliosis (6). A history of VKH disease therefore provides a distinctive immunological context in which to interpret pigmentary changes after pathway-specific treatment.

Here, we report a patient with prurigo nodularis and VKH disease who developed a psoriasiform eruption after a single 600-mg loading dose of dupilumab, followed by marked depigmentation at the preceding inflammatory sites. The spatial relationship among the pre-existing prurigo nodules, psoriasiform lesions, and subsequent depigmentation was particularly notable.

## 2 Case description

A 70-year-old Japanese man was referred for severe generalized pruritus and persistent nodular lesions. He had been diagnosed with atopic dermatitis approximately 20 years earlier and treated mainly with topical corticosteroids. About seven years before referral, the lesions became chronic and nodular, evolving clinically into prurigo nodularis. Pruritus worsened during the month before referral despite an oral corticosteroid/antihistamine combination. The overall clinical course is summarized in **Table 1**.

**Table 1:** Clinical timeline of the patient.

Time	Clinical course and intervention
0 M	Initial dermatology visit for severe prurigo nodularis. More than 100 pruriginous nodules were present, corresponding retrospectively to IGA PN-S 4 (severe), with baseline PP-NRS >7. A baseline skin biopsy was performed.
0.5 M	A single 600-mg loading dose of dupilumab was administered.
1 M	At follow-up approximately 2 weeks after dupilumab administration, the patient presented with a rapidly generalized psoriasiform eruption, largely sparing the pre-existing prurigo nodules. Several pre-existing nodules appeared mildly flatter, and excoriated or eroded areas showed partial epithelialization. Dupilumab was discontinued, and a second skin biopsy was performed.
1.5 M	Oral cyclosporine was initiated at 100 mg/day.
3.5 M	Cyclosporine was reduced to 100 mg every other day.
6 M	Cyclosporine was discontinued; topical treatment was continued.
7 M	Marked depigmentation was clearly documented photographically, approximately 6 months after dupilumab administration. Poliosis of the eyelashes and temporal scalp appeared more prominent during follow-up.
19 M	The inflammatory skin disease remained stable with topical treatment alone. Follow-up at our department ended, and the patient was referred to a local dermatologist.

M, months after the initial dermatology visit; IGA PN-S, Investigator’s Global Assessment for Prurigo Nodularis-Stage; PP-NRS, Peak Pruritus Numerical Rating Scale.

His medical history included Vogt-Koyanagi-Harada disease, diabetes mellitus, hypertension, gout, and cataract. He had no known history of psoriasis before dupilumab administration. VKH had been diagnosed approximately 20 years earlier at another hospital, but detailed historical records were unavailable; therefore, VKH activity at the time of dupilumab initiation could not be formally assessed. Baseline photographs showed poliosis of the eyelashes and temporal scalp.

At presentation, more than 100 excoriated and hyperkeratotic erythematous-brown nodules and papules were distributed over the trunk and extremities (**Figure 1a**), corresponding retrospectively to an Investigator’s Global Assessment for Prurigo Nodularis-Stage (IGA PN-S) score of 4 (severe). The baseline PP-NRS was >7. Biopsy of a representative nodule showed marked epidermal hyperplasia compatible with prurigo nodularis (**Figure 1b**). Serum total IgE was 2,464 IU/mL, thymus and activation-regulated chemokine (TARC) was 4,912 pg/mL, and peripheral eosinophils were 1.3%.

**Figure 1:**
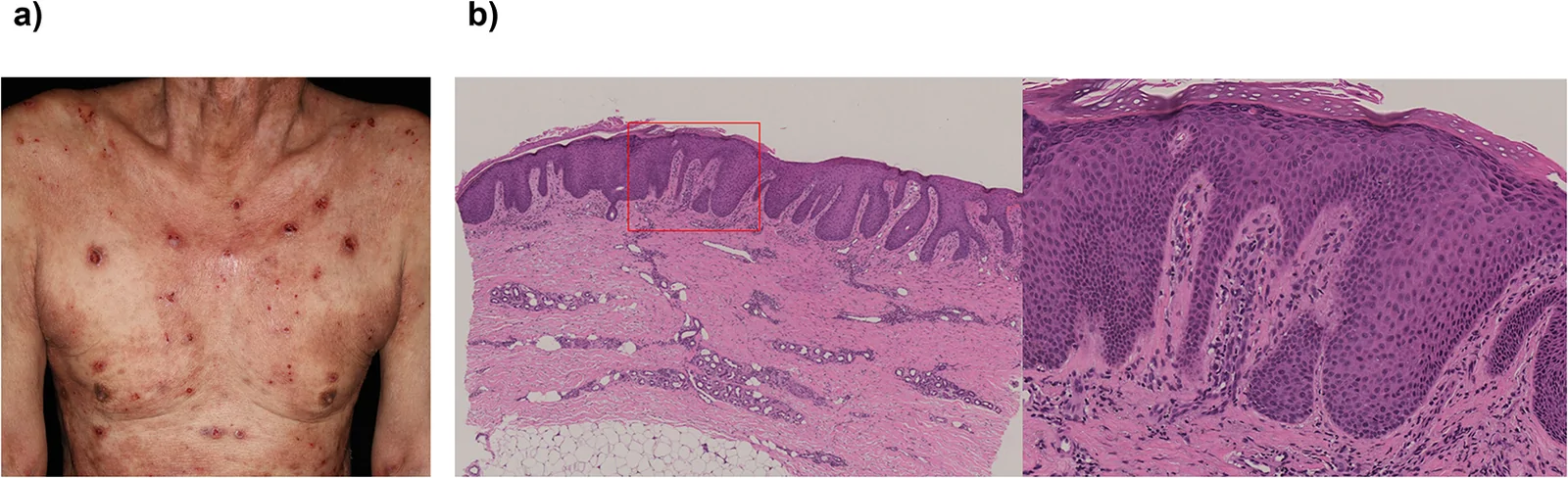
Clinical and histopathological findings before dupilumab treatment. **(a)** Baseline clinical presentation showing multiple excoriated pruriginous papules and nodules distributed over the trunk. **(b)** Hematoxylin and eosin staining of a pre-treatment lesion showing epidermal hyperplasia with chronic inflammatory changes compatible with prurigo nodularis. The boxed area in the low-power image is shown at higher magnification on the right.

Dupilumab was initiated for severe prurigo nodularis as a single 600-mg loading dose. At follow-up approximately two weeks later, the patient presented with a rapidly generalized psoriasiform eruption that developed predominantly between and around the pre-existing prurigo nodules, many of which were relatively spared (**Figure 2a**). Several pre-existing nodules appeared mildly flattened, and excoriated or eroded areas showed partial epithelialization.

**Figure 2:**
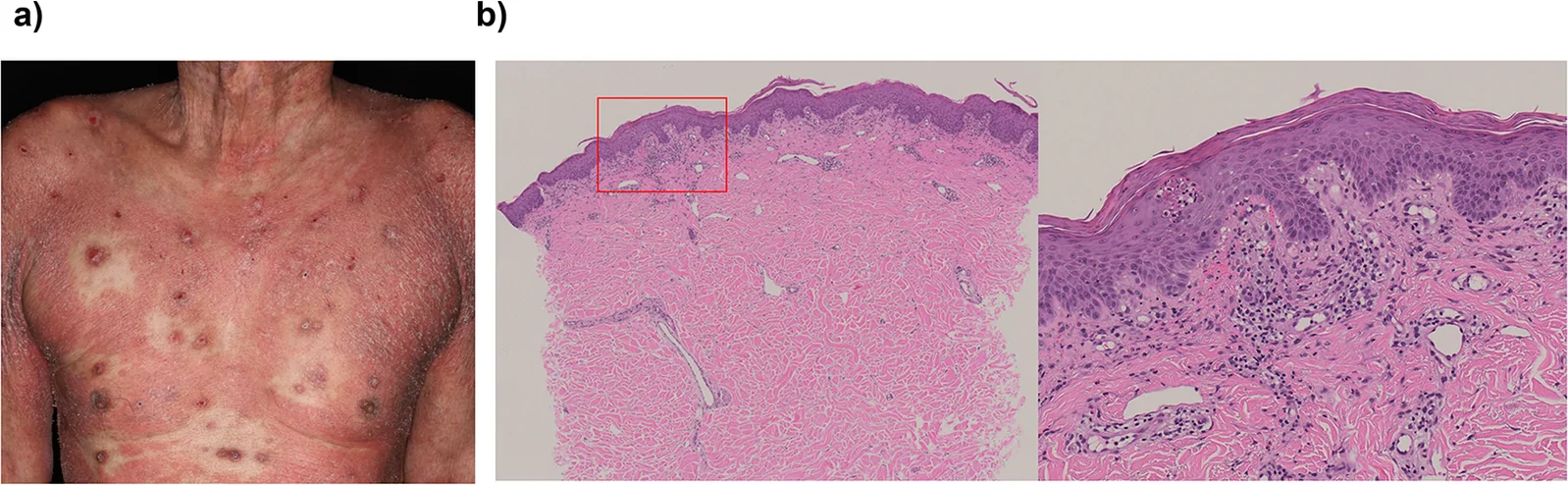
Psoriasiform eruption after dupilumab administration. **(a)** At follow-up approximately two weeks after a single 600-mg loading dose of dupilumab, the patient presented with a rapidly generalized erythematous psoriasiform eruption on the trunk in a striking distribution that largely spared the pre-existing pruriginous nodules. Several pre-existing nodules appeared mildly flatter, and excoriated or eroded areas showed partial epithelialization. **(b)** Hematoxylin and eosin staining of a newly developed plaque showing irregular epidermal hyperplasia with hyperkeratosis and parakeratosis, loss of the granular layer, focal neutrophilic infiltration, and small intraepidermal neutrophilic pustules, including a subcorneal neutrophilic collection suggestive of a Munro microabscess. Superficial perivascular inflammatory infiltrates included lymphocytes, histiocytes, neutrophils, and eosinophils with vascular dilatation; mild spongiosis, focal vacuolar alteration at the dermoepidermal junction, and rare dyskeratotic keratinocytes were also present. The boxed area in the low-power image is shown at higher magnification on the right.

Dupilumab was discontinued, and biopsy of a newly developed plaque was performed (**Figure 2b**). Histopathological examination showed irregular epidermal hyperplasia with hyperkeratosis and parakeratosis, loss of the granular layer, focal neutrophilic infiltration, and small intraepidermal neutrophilic pustules. Mild spongiosis, focal vacuolar alteration at the dermoepidermal junction, eosinophils, and rare dyskeratotic keratinocytes were also observed. Although regular psoriasiform acanthosis was not prominent, the clinicopathological findings were considered compatible with a treated or drug-associated psoriasiform eruption.

Oral cyclosporine was initiated at 100 mg/day, subsequently tapered, and discontinued at 6 months. At 7 months after the initial visit (approximately six months after dupilumab administration), extensive depigmented macules and patches were clearly documented on the trunk at sites corresponding to the preceding psoriasiform eruption (**Figure 3a**).

**Figure 3:**
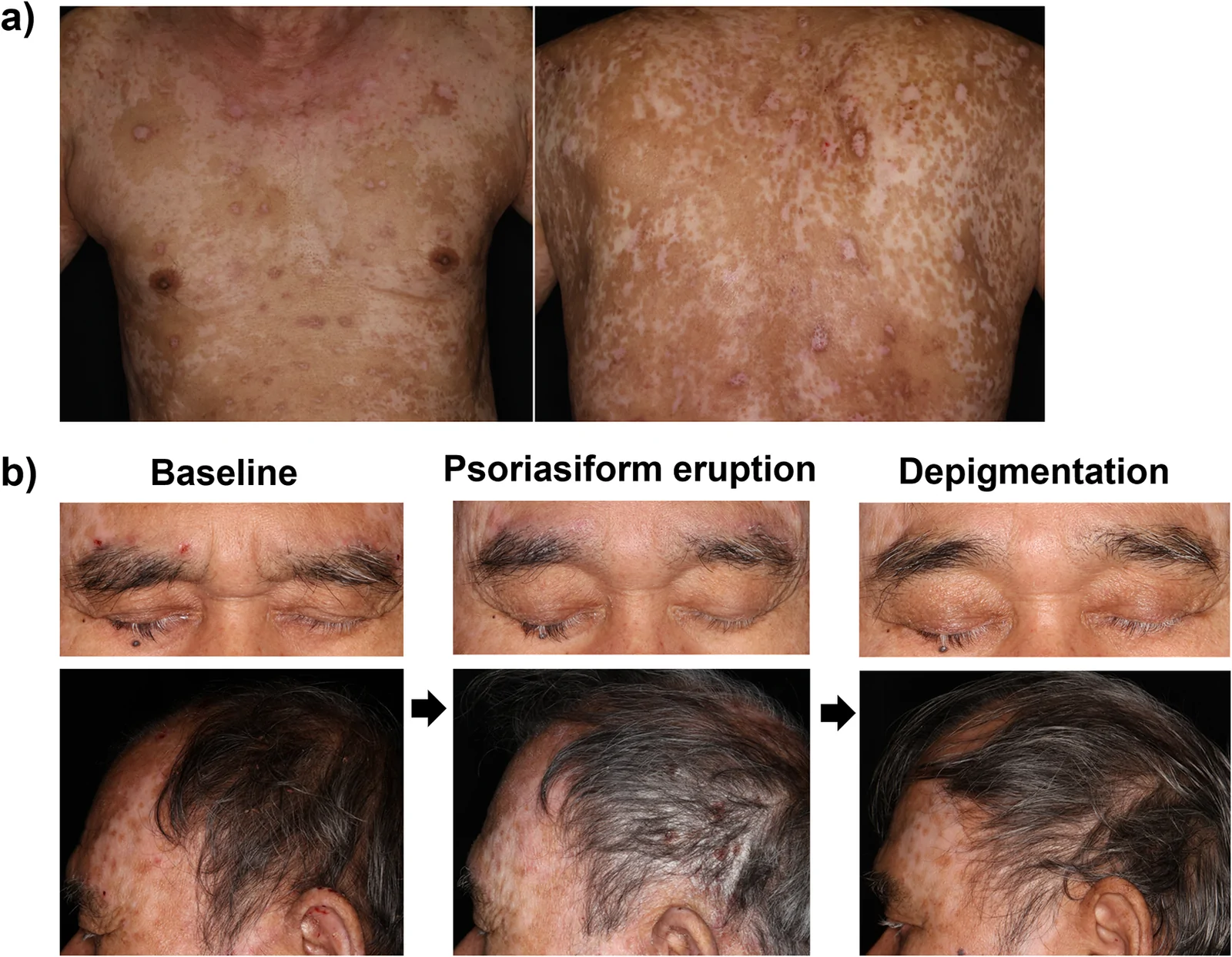
Depigmentation and longitudinal changes in poliosis during follow-up. **(a)** At 7 months after the initial visit (approximately six months after dupilumab administration), marked mottled depigmentation was documented over the trunk at sites previously affected by the psoriasiform eruption. The change was clinically considered severe post-inflammatory hypopigmentation, although vitiligo remained an important differential diagnosis because of the patient’s history of Vogt-Koyanagi-Harada disease. **(b)** Serial photographs of the eyelashes (upper row) and temporal scalp (lower row) at baseline, at the time of the psoriasiform eruption, and after development of extensive depigmentation. Poliosis was already present at baseline but appeared to become more prominent during follow-up, particularly in the left eyelashes and temporal scalp.

The pigmentary change was clinically considered severe post-inflammatory hypopigmentation; however, vitiligo remained an important differential diagnosis because VKH itself can cause vitiligo and poliosis and vitiligo has also been reported during dupilumab treatment (4–6). Poliosis of the eyelashes and temporal scalp, which had been present at baseline, appeared more prominent during follow-up (**Figure 3b**). These findings were not considered evidence of systemic VKH reactivation.

Wood lamp examination, dermoscopy, and biopsy or melanocyte-specific staining of the depigmented skin were not performed. We therefore use the descriptive term “depigmentation” rather than claiming definite dupilumab-induced vitiligo. The subsequent course of the depigmentation was not systematically documented. The inflammatory skin disease remained stable with topical therapy until follow-up ended at 19 months.

## 3 Discussion

This case shows three sequential cutaneous phenotypes in the same patient: chronic prurigo nodularis with a type 2 inflammatory background, a rapid psoriasiform eruption after IL-4/IL-13 blockade, and subsequent depigmentation at corresponding sites. The history of VKH disease and the presence of poliosis before dupilumab treatment are relevant because they indicate a pre-existing melanocyte-directed autoimmune background. However, the available clinical data do not establish reactivation of systemic VKH.

Dupilumab-associated psoriasis and psoriasiform eruptions are recognized paradoxical reactions. Suppression of IL-4/IL-13 signaling may reduce restraint on the IL-23/IL-17 axis, allowing a Th17-dominant phenotype to emerge in susceptible skin (1). Other Th17-associated inflammatory manifestations, including seronegative inflammatory arthritis and enthesitis/enthesopathy, have also been reported during dupilumab therapy, supporting the possibility that IL-4/IL-13 blockade may alter the balance between type 2 and IL-23/IL-17-associated immune pathways in susceptible individuals (7, 8). The short latency after a single 600-mg loading dose in our patient suggests that pre-existing immunological susceptibility may have contributed.

An unusual feature was the relative sparing of pre-existing prurigo nodules by the new plaques. Interestingly, comparison of serial photographs suggested that several pre-existing nodules became mildly flatter after the single dupilumab dose and that excoriated or eroded areas showed partial epithelialization, whereas the new psoriasiform eruption developed predominantly in the surrounding non-nodular skin. One possible explanation is that established prurigo nodules and the surrounding skin had different local immune set points. Chronic prurigo nodules are characterized by type 2 and neuroimmune dysregulation together with tissue remodeling (9, 10). In the nodules, IL-4/IL-13 blockade may therefore have primarily attenuated pre-existing type 2 inflammatory activity, consistent with the observed mild flattening and epithelialization. In contrast, the surrounding non-nodular skin may have had a different baseline immune susceptibility and may have been more permissive to the emergence of alternative inflammatory pathways after type 2 blockade. Given the patient’s melanocyte-directed autoimmune background associated with VKH, susceptibility to Th1/Th17-associated inflammation may have contributed to this differential response (7, 11). However, this interpretation remains hypothetical because no site-specific cytokine or cellular profiling was performed.

The subsequent depigmentation is also immunologically relevant. Vitiligo is driven largely by melanocyte-directed CD8 T cells and the IFN-γ-CXCL9/CXCL10-CXCR3 axis (2, 3). The therapeutic efficacy of topical ruxolitinib, a JAK1/JAK2 inhibitor, and oral upadacitinib, a JAK1-selective inhibitor, supports the importance of JAK-dependent signaling in vitiligo (12, 13).

Conversely, new-onset or worsening vitiligo has occasionally been reported during dupilumab treatment (4, 5). *In vitro* IL-4Rα blockade in stimulated T cells from patients with atopic dermatitis increased the proportion of IFN-γ-producing cells (14), providing limited experimental support for an immune shift away from type 2 signaling. However, these *in vitro* findings do not establish that dupilumab enhances IFN-γ signaling *in vivo* or promotes VKH activity. In our patient, the pre-existing VKH background and poliosis may indicate susceptibility to melanocyte-directed autoimmunity, but the proposed relationship between selective type 2 blockade and the subsequent depigmentation remains hypothetical because no cytokine, chemokine, melanocyte-specific immune profiling, or systematic assessment of systemic VKH activity was performed.

Severe post-inflammatory hypopigmentation remains an equally plausible explanation because the pigment loss closely followed the preceding inflammation. The absence of Wood lamp examination, dermoscopy, and melanocyte-specific evaluation prevents definitive distinction from vitiligo. The apparent increase in poliosis during follow-up is clinically relevant but is not diagnostic of vitiligo and does not establish systemic VKH reactivation. Accordingly, the event should not be labeled “dupilumab-induced vitiligo” on temporal association alone.

This case nevertheless raises a therapeutic question. In patients with type 2 inflammatory disease and established or suspected melanocyte-directed autoimmunity, broader cytokine-pathway modulation may be conceptually attractive. JAK inhibition can target IFN-γ signaling relevant to vitiligo while also modulating pathways involved in inflammatory dermatoses (12, 13). However, no comparative study has shown systemic JAK inhibition to be superior to dupilumab in such patients, and the present case does not establish that a JAK inhibitor would have prevented either the psoriasiform eruption or depigmentation. Treatment should therefore remain individualized.

A strength of this report is the well-documented temporal and spatial sequence supported by serial photographs and biopsies of the inflammatory lesions. Its main limitations are the lack of Wood lamp or melanocyte-specific evaluation of the depigmented lesions, absence of immune profiling, unavailable detailed historical ophthalmologic records regarding the original VKH manifestations and treatment, lack of systematic assessment of VKH activity during the present dermatologic follow-up, and the inability of a single case to establish causality. VKH itself also predisposes to vitiligo and poliosis.

In conclusion, this case illustrates a rapid psoriasiform eruption after dupilumab followed by site-matched depigmentation in a patient with prurigo nodularis and VKH disease. It highlights how pre-existing autoimmune context and local tissue state may influence inflammatory phenotypes emerging after selective cytokine blockade. The temporal relationship is hypothesis-generating rather than evidence of dupilumab-induced VKH reactivation or vitiligo, and further studies are needed to clarify the role of broader pathway-directed approaches in patients with melanocyte-directed autoimmunity.

## Data Availability

The original contributions presented in the study are included in the article/supplementary material. Further inquiries can be directed to the corresponding author.
